# A novel risk score system for assessment of ovarian cancer based on co-expression network analysis and expression level of five lncRNAs

**DOI:** 10.1186/s12881-019-0832-9

**Published:** 2019-06-10

**Authors:** Qian Zhao, Conghong Fan

**Affiliations:** Department of Gynecology & Obstetrics, Chengdu Women’s & Children’s Central Hospital, No.1617 Riyue Avenue, Chengdu, 610091 Sichuan Province China

**Keywords:** Ovarian cancer, LncRNAs, Co-expression network, Gene expression, Risk assessment tool, Functional enrichment analysis

## Abstract

**Background:**

Ovarian cancer (OC) is the most deadly gynaecological cancer, contributing significantly to female cancer-related deaths worldwide. Improving the outlook for OC patients depends on the identification of more reliable prognostic biomarkers for early diagnosis and survival prediction. The various roles of long non-coding RNAs (lncRNAs) in OC have attracted increasing attention. This study aimed to identify a lncRNA-based signature for survival prediction in OC patients.

**Methods:**

RNA expression data and clinical information from a large number of OC patients were downloaded from a public database. These data were regarded as a training set to construct a weighed gene co-expression network analysis (WGCNA) network, mine stable modules, and screen differentially expressed lncRNAs. The prognostic lncRNAs were screened using univariate Cox regression analysis and the optimal prognosis lncRNA combination was screened using a Cox-PH model. The finalised lncRNA combination was used to construct the risk score system, which was validated and assessed for effectiveness using other independent datasets. Further functional pathway enrichment was performed using gene set enrichment analysis (GSEA).

**Results:**

A co-expression network was constructed and four stable modules with OC-related biological functions were obtained. A total of 19 lncRNAs significantly related to prognosis of ovarian cancer were obtained using univariate Cox regression analysis, and the 5 prognostic signature lncRNAs GAS5, HCP5, PART1, SNHG11, and SNHG5 were used to establish a risk assessment system. The reliability of the prognostic scoring system was further confirmed using validation sets, which indicated that the risk assessment system could be used as an independent prognostic factor. Pathway enrichment analysis revealed that the network modules related to the above five prognostic genes were significantly associated with cell local adhesion, cancer signaling pathways, JAK-STAT signalling, and endogenous cell receptor interaction.

**Conclusions:**

The risk score system established in this study could provide a novel reliable method to identify individuals at high risk of OC. In addition, the five prognostic lncRNAs identified here are promising potential prognostic biomarkers that could help to elucidate the pathogenesis of OC.

## Background

Ovarian cancer (OC) is the most deadly gynaecological cancer and a primary cause of female cancer-related deaths worldwide, with recurrent OC being incurable in almost all cases [1, 2]. Due to the mild or absent signs and symptoms during early stages and the lack of reliable early detection tests, OC is usually diagnosed at the late stages, leading to poor prognosis and a five-year survival rate of only 30% [3, 4]. Therefore, a deeper understanding of its regulatory machinery at the molecular level is critical to identify reliable prognostic biomarkers for early diagnosis and survival prediction in OC patients.

Long non-coding RNAs (lncRNAs) are a group of non-coding RNAs longer than 200 nucleotides. Growing bodies of evidence have demonstrated the involvement of lncRNAs in OC, with at least 56 OC-related lncRNAs having been identified so far [4]. LncRNAs exhibit multiple biological functions during the various stages of OC development, and their deregulated expression is closely associated with OC early diagnosis, prognosis and response to chemotherapy [4–9]. Some lncRNAs, such as NEAT1 and GAS5, have been identified as clinical prognostic biomarkers, and are being used as potential therapeutic targets for OC [10–12]. Although previous studies have made remarkable progress, the prognostic roles of lncRNAs in OC and the related underlying mechanisms remain poorly characterized. Further research efforts are needed to identify, characterize, and elucidate the detailed functions of lncRNAs at the molecular level, and to identify more lncRNAs related to the prognosis of OC.

Recently, models for disease risk prediction and subsequent prognostic evaluation have attracted increasing interest from researchers, as it has become clear that the pathogenesis of most diseases is mediated by multiple, rather than single genes [13–16]. Risk assessment tools can help to estimate the probability that an individual with a given set of risk factors will develop a disease of interest, as well as detect high-risk populations for a given disease [15, 16]. So far, such risk assessment tools have been widely applied to the clinical prediction of various cancers [15, 17, 18], resulting in the identification of several expression-based lncRNA signatures as risk assessment tools for OC [3, 6, 9, 19]. However, as the risk assessment tools for OC are still limited, more research is necessary to more exhaustively establish a set of reliable tools for risk prediction.

In the present study, RNA expression data and clinical information from a large number of OC patients were downloaded from a public database, and a co-expression network was built to excavate network modules with OC-related biological functions. LncRNAs in stable functional modules were identified as important factors associated with OC. Combined with the clinical survival and prognostic information of OC samples; we identified a molecular lncRNA combination, which was significantly associated with OC prognosis. Based on these prognostic-related lncRNAs, a prognostic risk assessment model was constructed to identify significant differences between high-risk and low-risk prognostic samples. The reliability of the model was validated using the clinical survival and prognostic information of OC samples in independent validation datasets.

## Methods

### Data sources

The mRNA-seq expression profiles of OC in the training dataset were downloaded from the TCGA database (https://portal.gdc.cancer.gov/). A total of 419 ovarian cancer samples were detected by the Illumina HiSeq 2000 RNA sequencing platform. For the validation datasets, we first used the key word “ovarian cancer” to search all publicly uploaded expression data in the NCBI GEO (http://www.ncbi.nlm.nih.gov/geo/) database. The following criteria were then used for inclusion into the single validation datasets: 1) all data contained gene expression profiles; 2) detection objects were solid tumour tissues from patients with OC, exclusive of blood and cell lines; 3) expression profiles were all from human subjects; and 4) the sample number was no less than 40. The two final datasets GSE32062 and GSE17260 satisfied all the requirements, and included 260 and 100 samples, respectively.

### Data pre-processing

The downloaded data were pre-processed before further analysis. For the data downloaded from the TCGA database, normalisation was performed using the quantile standardization method of pre-process Core version 1.40.0 package [20] in R3.4.1 language (http://bioconductor.org/packages/release/bioc/html/preprocessCore.html). Next, lncRNAs were annotated using Ref_seq and Transcript_ID of the annotation platform and aligned by Blast to human genome sequences (GRCh38 version) using Clustal2 [21] (http://www.clustal.org/clustal2/). Finally, lncRNAs and their corresponding expression values were calculated [19].

For the expression profiles with CEL original format, data were converted into expression values using the oligoversion 1.41.1 package [22] in R3.4.1 language (http://www.bioconductor.org/packages/release/bioc/html/oligo.html). Missing data were supplemented with median values, followed by background correction using MAS methods and normalisation using quantile methods. For the expression profiles with. TXT original format, data were log_2_ transformed and normalised using the median standardization method in limma version 3.34.0 [23] in R3.4.1 language (https://bioconductor.org/packages/release/bioc/html/limma.html).

### Selection of stable modules using WGCNA

Weighed gene co-expression network analysis (WGCNA), a bioinformatics algorithm for construction of co-expression networks, is commonly used to identify modules associated with diseases and consequently screen important pathogenic mechanisms or potential therapeutic targets [24]. A co-expression network was constructed using the WGCNA version 1.61 package [25] in R3.4.1 language (https://cran.r-project.org/web/packages/WGCNA/index.html) based on the TCGA datasets.

WCGNA analysis needed to satisfy the prerequisite of scale-independent network distribution. Hence, the appropriate weight parameter β (power) of the adjacency matrix needed to be selected to ensure the constructed co-expression network approached the scale-free distribution to the greatest extent. With 1~20 of β value ranges, the linear model was established by logarithms of the adjacency degree of a node (log k) and the appearance probability of this node [log(p(k))]. The β parameters were the square values of R coefficients. A higher R^2^ value indicated that the network was closer to the scale-independent distribution. The β (power) value when R^2^ was approximately 0.9 for the first time was finally chosen for further analysis. All genes were ranked according to their expression values using a rank function. Then, the correlation between each pair of datasets of the three expression profiles (TCGA, GSE32062 and GSE17260) was evaluated using the verboseScatterplot function and RNA adjacency matrix to construct an RNA correlation matrix. This matrix was used as the basis to build the hierarchical clustering tree using the criteria of a cut height of 0.99, and the involvement of at least 200 RNAs per module. Finally, the userListEnrichment function was used to identify stable modules associated with OC using the criteria of a Z-score greater than 5. The lncRNAs in the stable modules were defined as those significantly associated with OC.

### Construction of the risk prediction model

The lncRNAs associated with prognosis were further identified by univariate Cox regression analysis of the survival package in R3.4.1 language. A *P* value less than 0.05 obtained by the log-rank test was chosen as the threshold for identification. Next, the optimal combinations of prognostic-related lncRNAs were screened using the Cox-Proportional Hazards (Cox-PH) model [26] based on L1-penalised regularization regression algorithm of the penalised package [27] in R3.4.1 language (http://bioconductor.org/packages/penalised/). The optimized parameter “lambda” in the screening model was obtained by 1000 cyclic calculations using a cross-validation likelihood (CVL) algorithm. Based on the regression coefficient of each lncRNA in the optimal lncRNA combination, lncRNA expression weighted by these coefficients was used to establish the risk prediction model, from which the risk score (RS) of each sample was obtained. The RS calculation formula was as follows:

$$ \mathrm{RS}=\upbeta \mathrm{lncRNA}1\times \mathrm{exprlncRNA}1+\cdots +\upbeta \mathrm{lncRNA}\mathrm{n}\times \mathrm{exprlncRNA}\mathrm{n} $$

### Validation of the risk prediction model

In order to validate the risk prediction model, GSE32062 and GSE17260 were used as single validation sets. Datasets meeting the following criteria were selected as independent validation datasets: 1) all data contained gene expression profiles; 2) detection objects were solid tumour tissues from patients with OC, exclusive of blood and cell lines; 3) expression profiles were all from human subjects; 4) samples had prognostic clinical information and 5) the sample number was no less than 50. This led to the selection of the three microarray datasets GSE49997, GSE26712, and GSE31245 containing 204, 185, and 58 samples, respectively. We also downloaded the mRNA-seq expression profiles of cervical cancer (CESC) samples with the corresponding clinical information data from TCGA to further validate the efficiency of the risk prediction model. Of 309 samples that were downloaded, 293 had clinical information available and were therefore used for validation. The risk-score model was used to assess significant prognostic differences between high and low risk groups, with the results determining the stability of this established model.

### Analysis of important lncRNA relevant pathways

Gene sets were isolated from the modules that contained the optimal lncRNAs significantly associated with OC prognosis. The GSEA (gene set enrichment analysis) method [28] (http://software.broadinstitute.org/gsea/index.jsp) was then used to identify KEGG pathways enriched in these optimal lncRNA-related gene sets. The GSEA-based pathway enrichment analysis was performed on each lncRNA counter-gene in the lncRNA-mRNA network. Three key statistical values were used in this analysis. The first of these, the enrichment score (ES), is the original result of the GSEA analysis, reflecting the degree of enrichment of one functional gene set locating at the anterior or posterior of this gene sequence after all the hybridization data are ordered. The fundamental calculation principle is to scan the collating sequence; when a gene of this set is prepared, the ES value will increase, otherwise it will decrease. The second key statistical value is the normalised enrichment score (NES) that results from standardized processing of ES values. The final statistical value is the nominal *P* value, which describes the statistical significance of the enrichment scores of a functional gene subset; a smaller P value indicates a better degree of gene enrichment. When the NES absolute value increases, the *P* value will spontaneously decrease, suggesting a higher degree of enrichment and a higher significance of the result. In this study, a P value less than 0.05 was chosen as the threshold to screen KEGG pathways that were significantly enriched in the relevant module genes.

## Results

### Screening of stable modules significantly correlated with OC using WGCNA

**Fig. 1:**
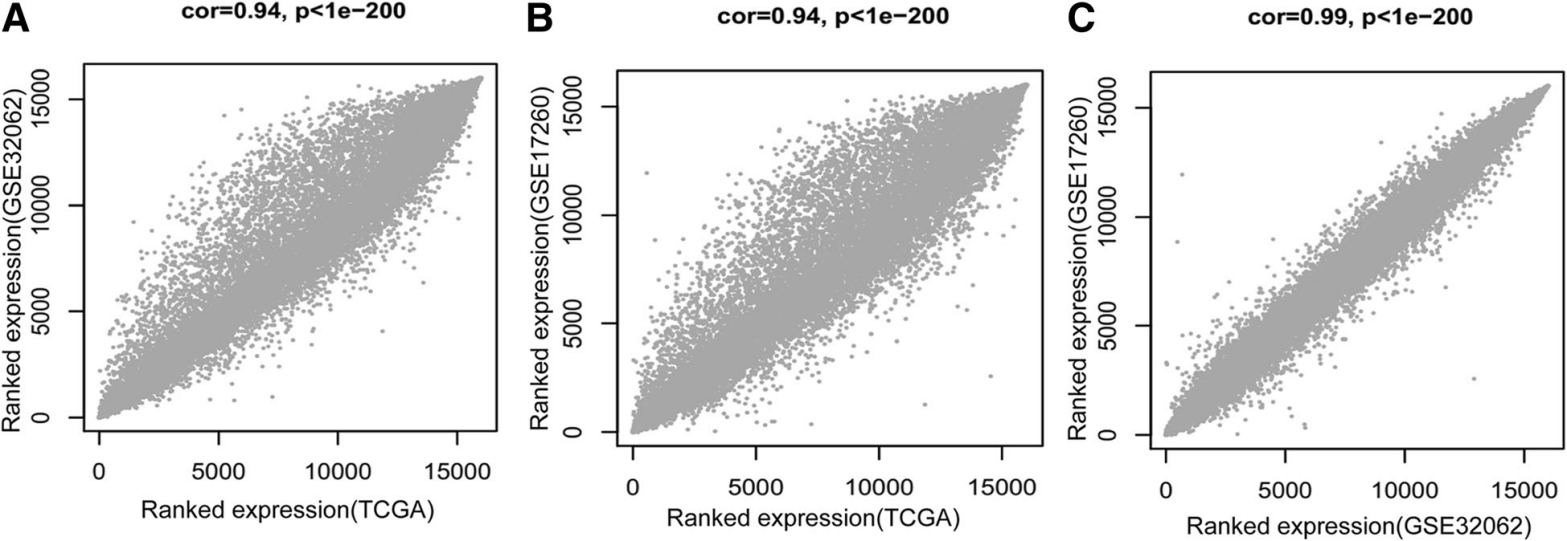
Correlation of expression levels between each pair of the three datasets; TCGA, GSE32062 and GSE17260. The graphs (from left to right) show the comparisons: TCGA-GSE32062 (**a**), TCGA-GSE17260 (**b**) and GSE32062-GSE17260 (**c**)

One dataset from TCGA and two datasets from NCBI GEO (GSE32062 and GSE17260) containing RNA expression data for OC were used for analysis. We identified 13,251 mRNAs and 646 lncRNAs overlapping in the three datasets (TCGA, GSE32062, and GSE17260). TCGA expression data was used as the training set and the remaining two datasets were regarded as validation datasets. First, the consistency of the expression values of the overlapped RNA in the three datasets was checked. The results indicated that the correlation distribution of RNA expression levels between each pair of datasets was higher than 0.9 with *P* values less than 1e^− 200^, suggesting that all three sets displayed significant positive correlation (Fig. 1).

**Fig. 2:**
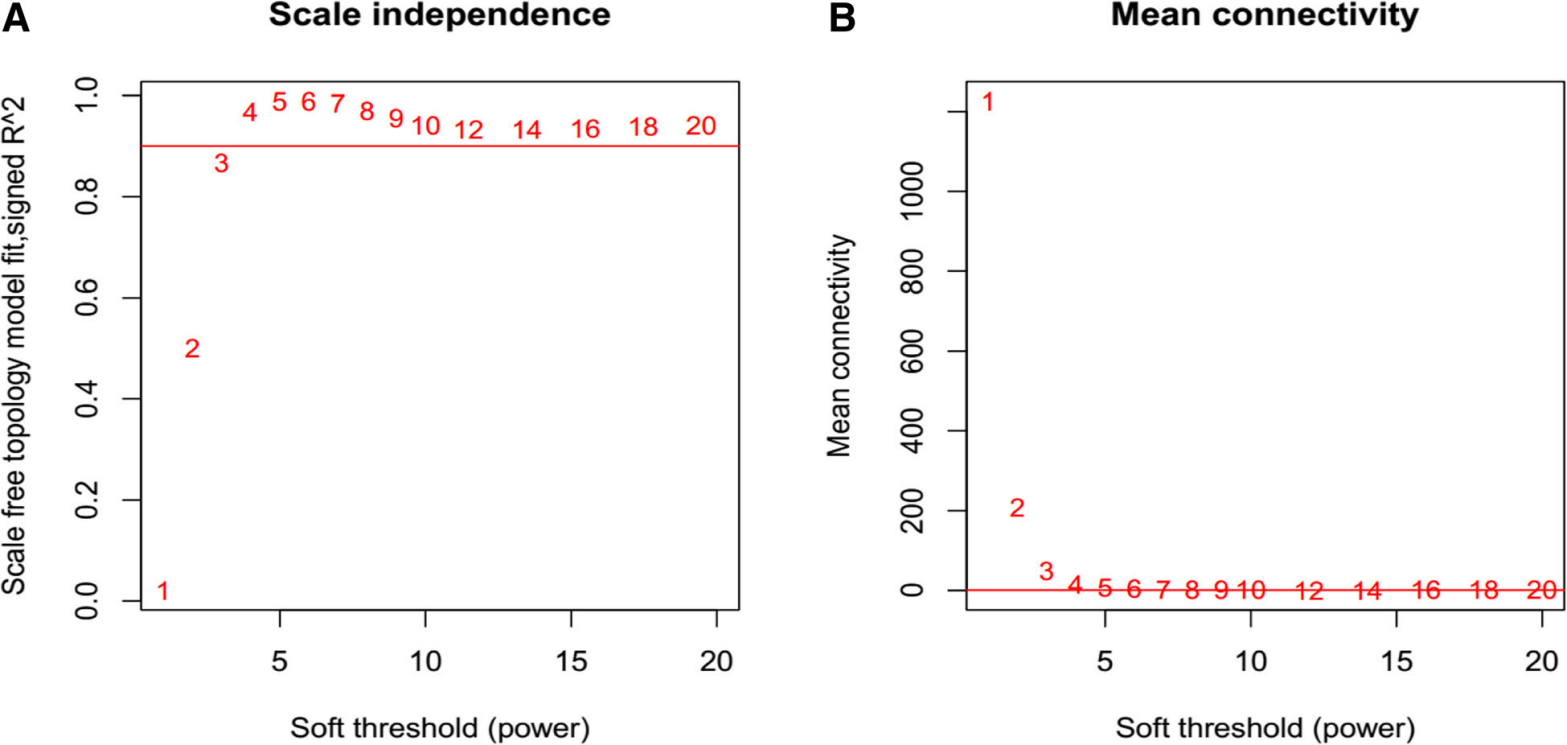
Selection graphs of the weight parameter β in the adjacency matrix (**a**) and schematic diagram of the average connectivity of RNA under various power parameters (**b**). The horizontal and vertical axes represent the weight parameter power and the square values (R^2) of correlation coefficients between log (k) and log (p(k)) in the corresponding network, respectively. The red line represents the standard line when R^2 reached a value of 0.9

WGCNA analysis was further conducted to screen stable modules associated with OC. The β (power) value of 3 was chosen as this was its value when R^2 was approximately 0.9 for the first time (Fig. 2a). This value of the β parameter not only ensured that the network connection was close to the scale-free distribution, but was also the minimum threshold to give the curve a tendency to be smooth. When the β value was equal to 3, the RNA average connection degree in the network was 3 (Fig. 2b), conforming to the small-world network character in scale-independent networks.

**Fig. 3:**
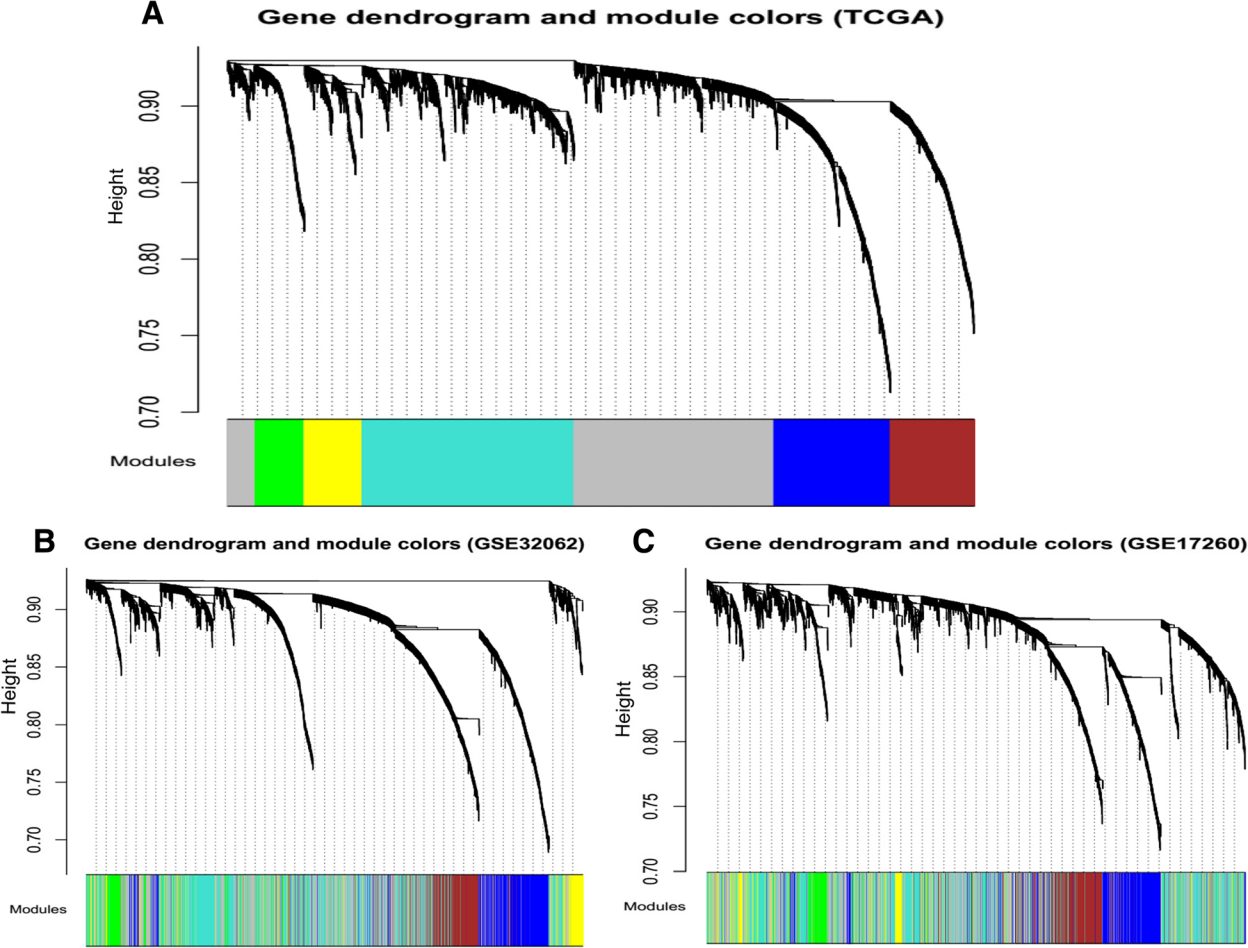
The tree views of RNA module division in the datasets TCGA (**a**), GSE32062 (**b**), and GSE17260 (**c**). Each colour represents one module

**Fig. 4:**
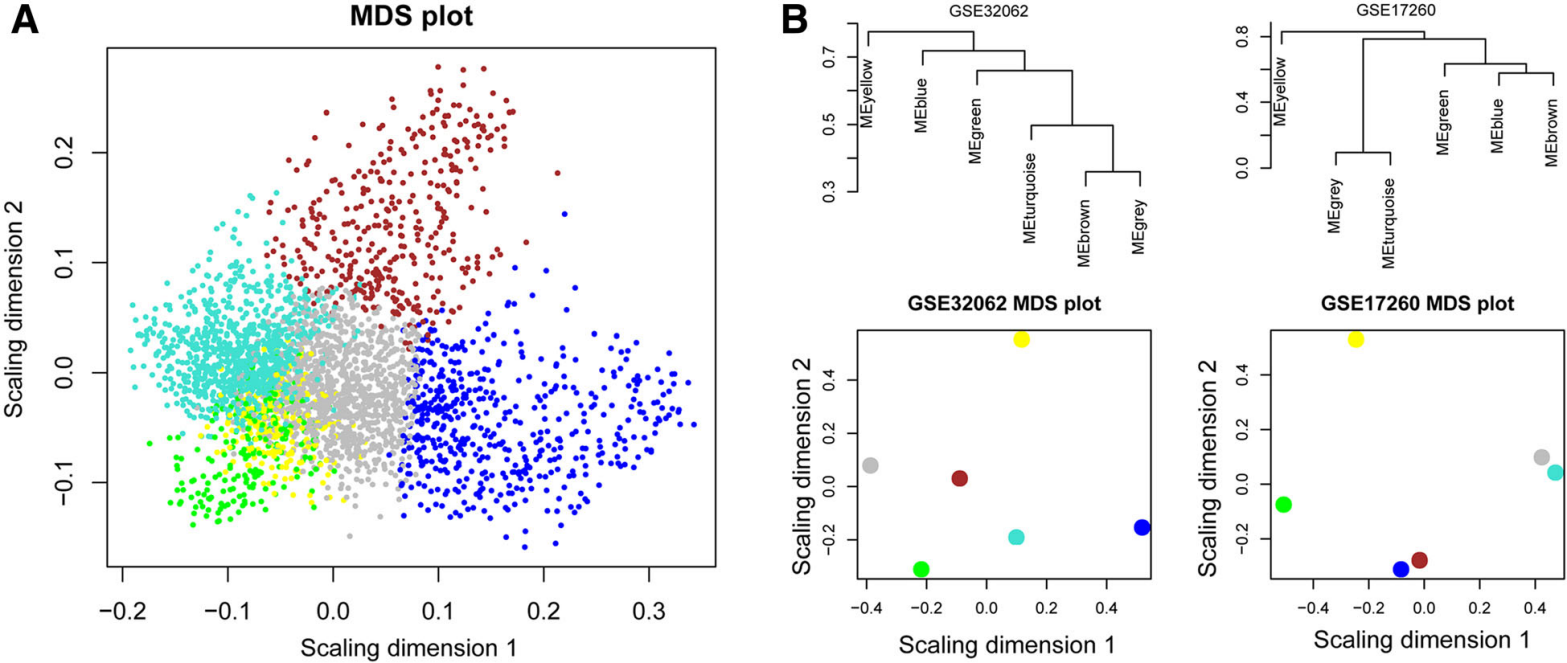
Multidimensional expansion map (MDS) of each gene located in a module of the TCGA dataset (**a**), and system cluster tree diagram of modules in datasets GSE32062 and GSE17260 (**b**). The X and Y axes represent the matching first and second principal components, respectively

An RNA adjacency matrix was constructed and a hierarchical clustering tree was built on basis of this matrix. Based on the criteria of a cut height equal to 0.99 and an involvement of at least 200 RNAs per module, six modules were identified; M1-blue, M2-brown, M3-green, M4-grey, M5-turquoise, and M6-yellow (Fig. 3a). The corresponding modules were partitioned in the single validation datasets GSE32062 (Fig. 3b) and GSE17260 (Fig. 3c), based on their inclusive RNA in the training set (TCGA) to evaluate the stability of modules selected from the training set. The results of the partition and correlation of modules in the TCGA dataset are displayed in Fig. 4. It was found that RNA in the same modules (i.e. dots with same colour) tended to aggregate together (Fig. 4a), indicating that RNA in the same module had similar expression. The clustering results of module RNA in the other two datasets indicated that the blue, brown and green modules exhibited characteristics of independent branches (Fig. 4b).

The results of stability analysis on the above six modules showed that the stability scores (preservation Z-score) of the blue, yellow, brown and green modules were all higher than 5, whereas those for the gray and turquoise modules were not. RNAs included in these four stable functional modules were likely associated with OC pathogenesis. Table 1 lists the functional annotation information of the six modules, showing that RNAs in the top four highly stable modules were mainly involved in cellular immune response, cell adhesion, sexual reproduction, and the cell cycle.

**Table 1:** Statistical information of module stability (preservation) and annotation in TCGA, GSE32062 and GSE17260 datasets

Module	Color	Module size	mRNA	lncRNA	Preservation Z-score	Module annotation
Module 1	blue	517	503	14	40.5499	Immune response
Module 2	brown	368	361	7	40.4322	Cell adhesion
Module 3	green	214	210	4	22.0548	Sexual reproduction
Module 4	grey	1990	1982	8	2.8158	Ion transport
Module 5	turquoise	920	915	5	4.6453	RNA metabolic process
Module 6	yellow	255	247	8	9.6416	Cell cycle

The first column is the number of module, the second column is the color corresponding to each module, the third column is the number of RNA in each module, the fourth column is the number of mRNA in each module, the fifth column is the number of lncRNA in each module and the sixth column is the preservation z-score corresponding to the stability of each module. The higher the z-score, the higher the stability of the module. Z-score between 5 and 10 means the module has stability and z-score > 10 means the module has high stability. The seventh column is the GO functions of each module

**Fig. 5:**
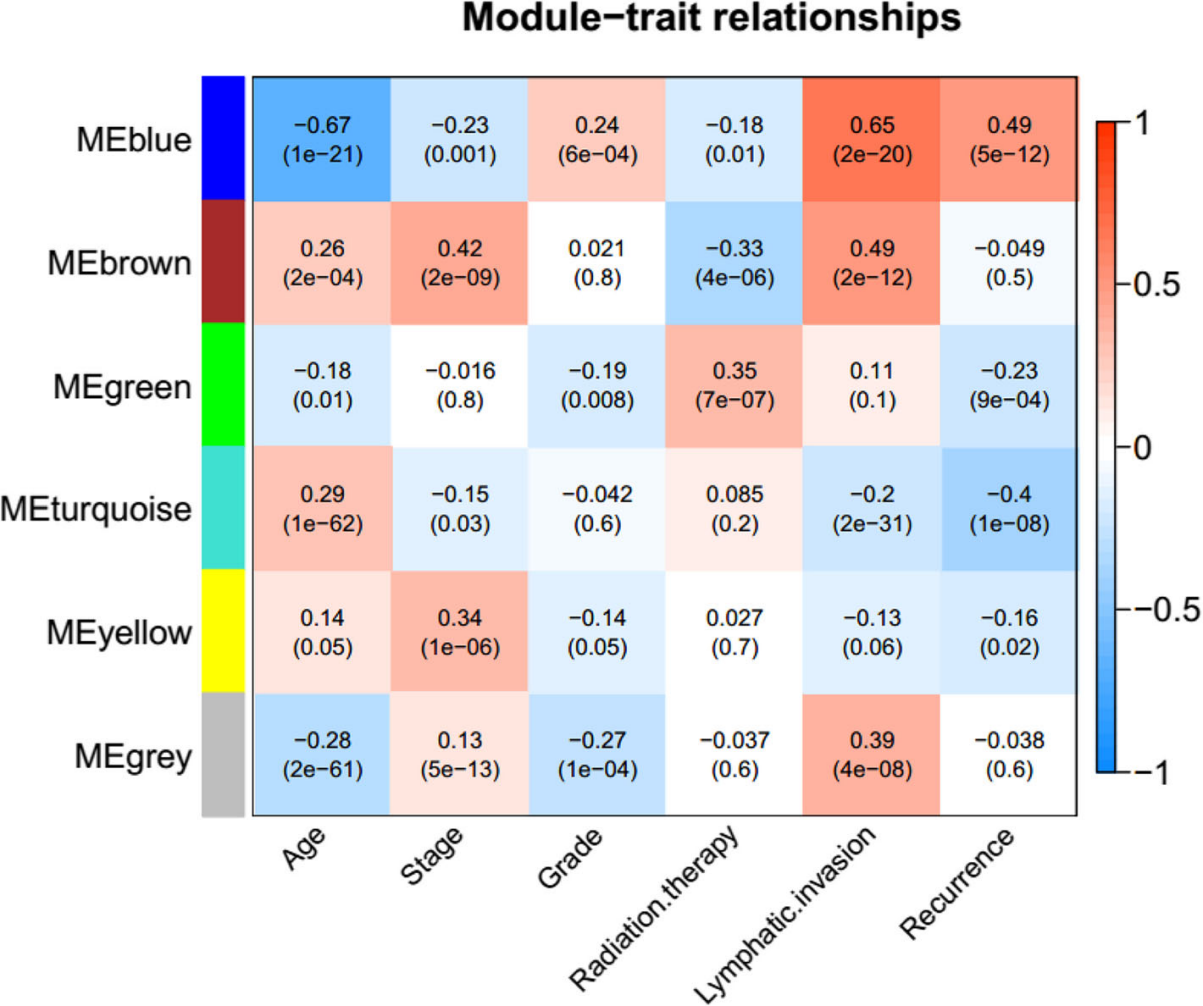
Correlation analyses between functional modules of RNAs in the TCGA dataset and their clinical attributes

The corresponding clinical information of samples in the TCGA dataset was integrated and the correlation between RNA obtained by partitioning in each module and clinical factors was calculated (Fig. 5). The results showed that the four highly stable RNA modules brown, yellow, blue and green were significantly correlated using OC data including stage, grade, radiotherapy, lymph node metastasis, and recurrence. Therefore, a total of 33 lncRNAs in the four highly stable modules which were enriched for specific biological functions and displayed significant relationships with clinical factors of OC were selected as important OC-related factors for subsequent analyses.

### Establishment and evaluation of risk assessment model

#### Selection of optimal lncRNA combinations

We used Cox univariate regression analysis (with the regression threshold P set at 0.05) to evaluate the expression levels of the 33 lncRNAs within the stable modules of the TCGA dataset with regard to the OC sample clinical prognosis information. This resulted in the identification of 19 prognostic related-lncRNAs (Table 2).

**Table 2:** List of prognostic related lncRNAs obtained by COX univariate regression analysis

LncRNA	*p* value	Module color
MYCNOS	< 0.01	yellow
SNHG11	< 0.01	blue
PART1	< 0.01	green
SNHG5	< 0.01	yellow
FAM182B	< 0.01	brown
DSCR9	0.01	brown
GAS5	0.01	yellow
FAM182A	0.01	brown
KCNQ1DN	0.01	blue
SNHG1	0.02	blue
HCG27	0.03	green
RFPL1S	0.03	blue
SNHG7	0.04	blue
DLEU2	0.04	brown
HOTAIR	0.04	blue
HCP5	0.04	blue
DLEU1	0.05	brown
SNHG9	0.05	yellow
MIAT	0.05	yellow
LOH12CR2	0.05	yellow

Next, we optimized and screened the 19 OC prognostic-related lncRNAs using a Cox-PH model based on an L1-penalised regularization regression algorithm in the penalised package, in which the value of the “lambda” parameter was obtained by 1000 cyclic calculations using a CVL algorithm. This resulted in an optimal prognosis combination containing 5 lncRNAs: GAS5, HCP5, PART1, SNHG11 and SNHG5 (Table 3).

**Table 3:** Coefficients of optimal prognostic related lncRNA

LncRNA	Coef	Hazard ratio	*p* value	Module color
GAS5	0.109959	1.538315	0.038	yellow
HCP5	−0.492573	0.875803	0.021	blue
PART1	−0.966947	0.839052	0.016	green
SNHG11	0.319810	1.27635	0.005	blue
SNHG5	−0.272969	0.943449	0.015	yellow

Note: Coef was the coefficient value calculated by Cox-PH regression model

#### Construction, evaluation and selection of risk prediction models

Based on the corresponding Cox-PH regression coefficients of the optimal combination of 5 lncRNAs obtained in the previous section, the prediction model of sample risk scoring was established as follows:

$$ \mathrm{RS}=(0.11)\times \mathrm{Exp}\mathrm{GAS}5+\left(\hbox{-} 0.49\right)\times \mathrm{Exp}\mathrm{HCP}5+\left(\hbox{-} 0.97\right)\times \mathrm{Exp}\mathrm{PART}1+(0.32)\times \mathrm{Exp}\ \mathrm{SNHG}11+\left(\hbox{-} 0.27\right)\times \mathrm{Exp}\ \mathrm{SNHG}5 $$

**Fig. 6:**
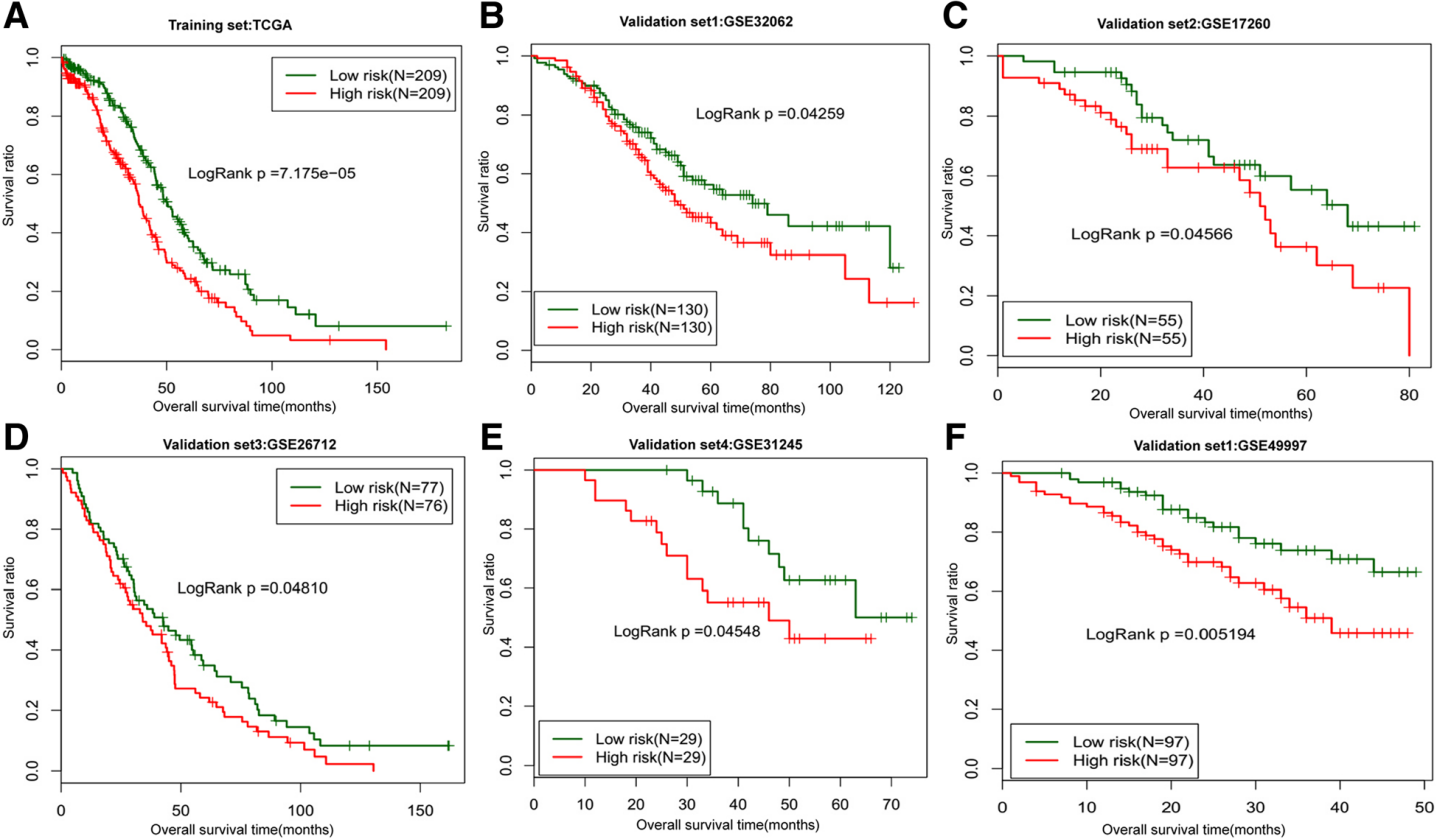
Kaplan-Meier survival curves of samples in training set TCGA (**a**), validation sets GSE32062 (**b**) and GSE17260 (**c**), and independent validation sets GSE26712 (**d**), GSE31245 (**e**), and GSE49997 (**f**) using the risk assessment model established by the 5 optimal lncRNAs. The green and red curves represent the low- and high-risk groups, respectively

**Fig. 7:**
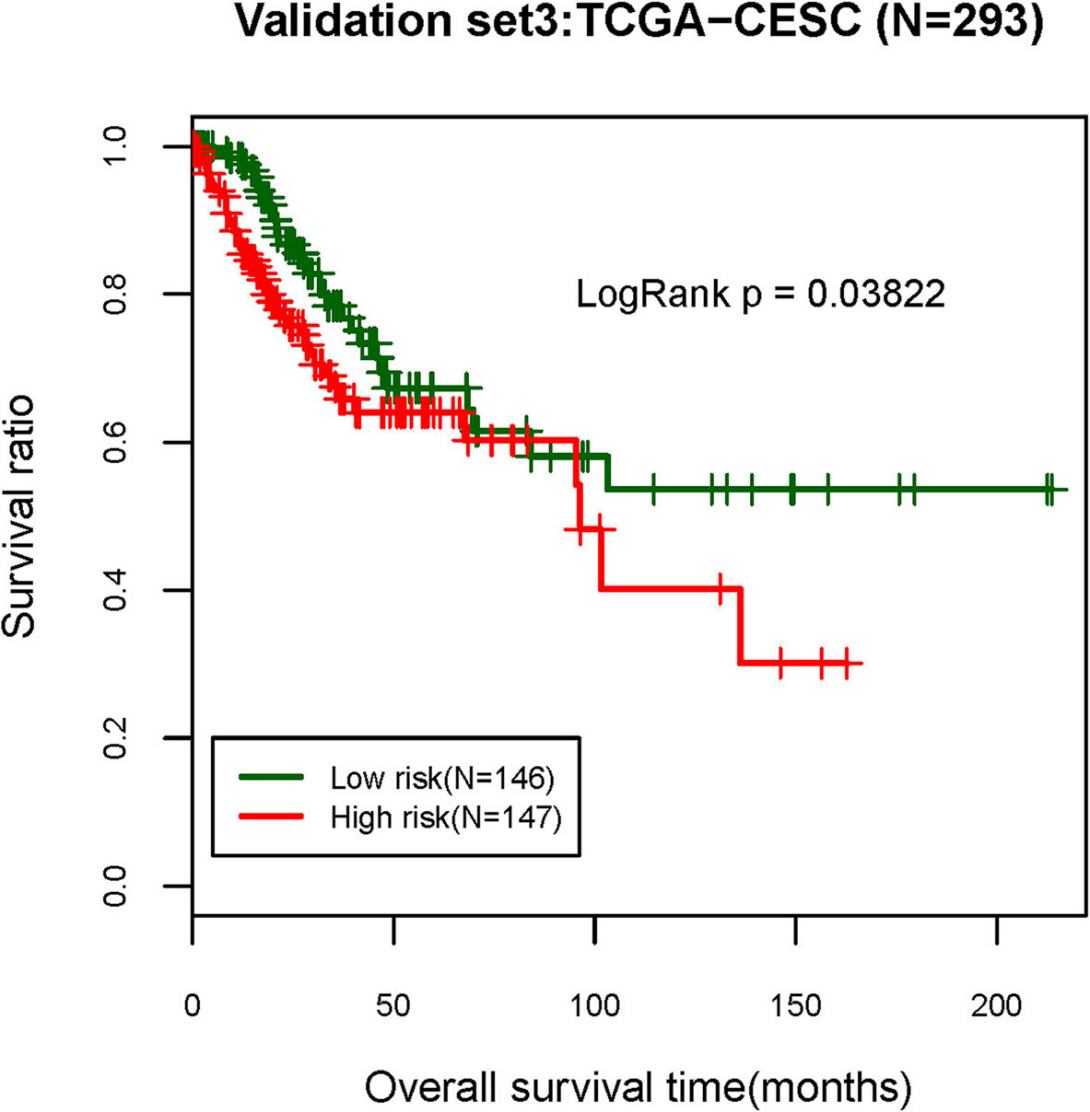
Kaplan-Meier survival curves of samples in validation set of cervical cancer from TCGA to validate the efficiency of the risk prediction model

The RS of each sample in the training set (TCGA) was calculated according to the constructed model. These samples were divided into high- and low-risk groups based on the scoring medians. Next, Kaplan-Meier survival curve analysis was used to examine the significant differences in survival time between the two sample groups. The result indicated that this model had an excellent distinguishing effect on the sample groups (*p* value < 0.01) (Fig. 6 and Fig. 7); therefore we used this model for subsequent analyses.

As shown in Fig. 7, the prediction model also displayed significant differentiation effects on samples of the GSE32062 and GSE17260 datasets included in the WGCNA analysis (*P* value < 0.05). The stability of this model was further validated by the independent validation datasets GSE49997, GSE26712, and GSE31245, as well as the CESC data from TCGA exclusive of any processing (including module discovering and survival analysis). The model displayed significant differentiation effects for all these validation sets, indicating a high robustness of this prediction model. Taken together, the above results show that the 5 lncRNAs comprising the key components of this risk prediction model (GAS5, HCP5, PART1, SNHG11, and SNHG5), were significantly associated with OC prognosis, and form a stable combination for distinguishing high- and low-risk prognostic samples, as well as other unprocessed samples.

### Network construction and functional pathway analysis

**Fig. 8:**
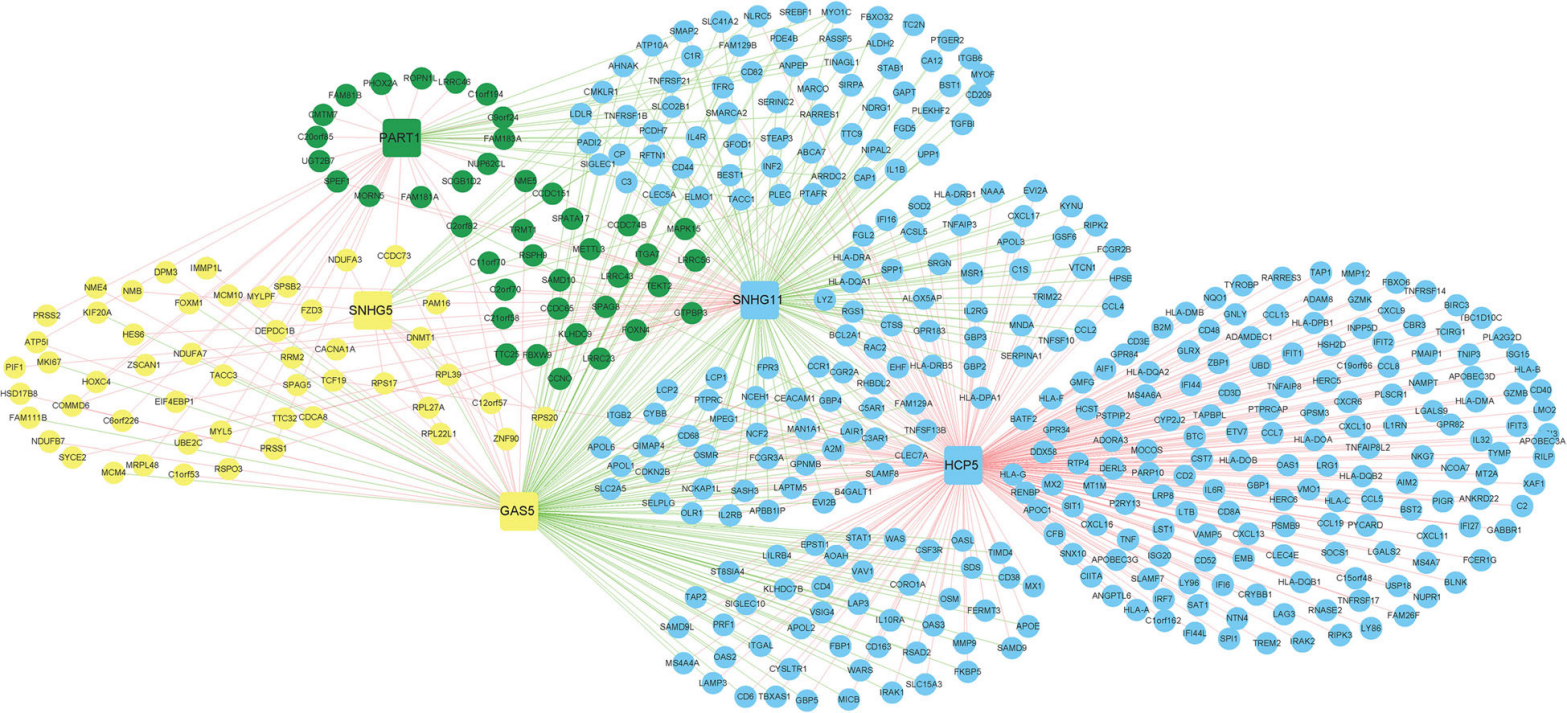
Graphs of lncRNA-mRNA networks corresponding to the blue, green, and yellow modules. The circular node represents mRNA, and the square node represents lncRNA. The colour of the node indicates that it belongs to the corresponding module, and the green and red connecting lines indicate negative and positive correlation, respectively

The previous analysis identified the 5 important OC-related lncRNAs; GAS5, HCP5, PART1, SNHG11, and SNHG5. These five lncRNAs were distributed in the blue, green, and yellow stable modules discovered by the WGCNA method. The mRNAs closely related to the expression of these five lncRNAs can be found in these modules, and may be the target genes of these key lncRNAs (Fig. 8).

GSEA was used to perform pathway enrichment analysis of these mRNAs. This showed that the network modules related to the 5 prognostic genes were significantly associated with cell local adhesion, cancer signalling pathways, JAK-STAT signalling, and endogenous cell receptor interaction (Table 4).

**Table 4:** KEGG pathways significantly related to blue, green and yellow modules

Module/lncRNA	Name	ES	NES	NOM p-val	FDR q-val	Gene
Yellow/GAS5, SNHG5	Pyrimidine Metabolism	−0.461	−1.349	< 0.001	< 0.001	*TYMP, UPP1, RRM2, NME4, NME5*
	Regulation Of Actin Cytoskeleton	0.365	1.167	0.002	0.006	*WAS, VAV1, ITGB2, ITGAL, NCKAP1L, ITGB6, MYLPF, ITGA7*
	Fc Gamma R Mediated Phagocytosis	0.388	1.153	< 0.001	0.006	*WAS, VAV1, FCGR3A, INPP5D, PTPRC, FCGR2A, FCGR2B,*
	JAK STAT Signaling Pathway	0.404	1.153	< 0.001	0.008	*CSF3R, IL2RG, IL6R, IL4R, IL10RA, OSM, OSMR, IL2RB*
	Ribosome	−0.971	−1.156	< 0.001	0.028	*RPS17, RPL39, RPL27A, RPL22L1, RPS20*
Blue/HCP5 SNHG11	Endocytosis	−0.328	−1.593	< 0.001	< 0.001	*HLA-B, HLA-C, HLA-A, HLA-F, TFRC, IL2RG, LDLR, HLA-G, IL2RB, SMAP2*
	Antigen Processing and Presentation	−0.271	−1.695	< 0.001	< 0.001	*CTSS, HLA-B, HLA-C, HLA-A, HLA-F, HLA-DMA, B2M, HLA-DPA1, HLA-DRB5, TAP2, TAP1, HLA-DPB1, HLA-DRA, HLA-DRB1*
	Lysosome	−0.664	−1.846	< 0.001	< 0.001	*CTSS, TCIRG1, LAMP3, LAPTM5, CD68*
	Pathways in Cancer	0.488	1.653	< 0.001	< 0.001	*CSF3R, RASSF5, FZD3, CDKN2B, MMP9, BIRC3*
	Focal Adhesion	0.435	1.432	< 0.001	< 0.001	*VAV1, MYLPF, ITGB6, ITGA7, BIRC3*
	Fc Gamma R Mediated Phagocytosis	−0.324	−1.127	< 0.001	0.049	*WAS, FCGR3A, INPP5D, RAC2, PTPRC, FCGR2A, FCGR2B, VAV1*
	JAK STAT Signaling Pathway	0.418	1.222	< 0.001	0.002	*CSF3R, IL2RG, IL6R, IL4R, IL10RA, OSM, OSMR, IL2RB*
	Pyrimidine Metabolism	−0.497	−1.451	< 0.001	< 0.001	*TYMP, UPP1, RRM2, NME4, NME5*
Green/PART1	Pyrimidine Metabolism	−0.530	−1.520	0.001	0.004	*RRM2, NME4, NME5, TYMP, UPP1*
	Fc Gamma R Mediated Phagocytosis	0.402	1.239	0.003	0.012	*VAV1, PTPRC, WAS, FCGR3A, FCGR2A, INPP5D, FCGR2B*
	B Cell Receptor Signaling Pathway	0.469	1.186	0.007	0.016	*VAV1, BLNK, INPP5D, FCGR2B*
	T Cell Receptor Signaling Pathway	0.528	1.117	0.015	0.033	*VAV1, PTPRC, CD3E, CD3D, TNF, CD8A, LCP2*
	Pathways in Cancer	0.320	1.247	0.004	0.013	*CDKN2B, CSF3R, MMP9, RASSF5, BIRC3*

## Discussion

OC is the most deadly gynaecological cancer and a primary cause of female cancer-related deaths worldwide, yet the regulatory machinery underlying OC development remains unclear. Increasing evidence has identified lncRNAs in multiple biological functions at various stages of OC development, and deregulated expression of lncRNA is closely associated with OC early diagnosis, prognosis, and response to chemotherapy [4–9]. To identify a novel lncRNA-based signature for predicting prognosis of OC patients, RNA expression profiling data and clinical survival prognosis information from a large number of OC patients were downloaded from the public database. A co-expression network was subsequently constructed and modules with OC-related biological functions were excavated.

As a bioinformatics algorithm for construction of co-expression networks, WGCNA is commonly used to identify modules associated with diseases and consequently screen important pathogenic mechanisms or potential therapeutic targets [24]. So far, gene modules associated with several cancers have been identified and validated through co-expression network analysis [29–31]. In the present study, six modules were obtained in the training set, four of which (blue, yellow, brown, and green) displayed high stability. The results of functional annotation analysis showed that RNAs in these four highly stable modules were mainly involved in cellular immune responses, cell adhesion, the cell cycle, and sexual reproduction, indicating their likely association with OC pathogenesis.

Prognostic genes are informative for cancer prognosis and treatment because of their potential as biomarkers, and can help to predict patients’ survival, as well as providing insights into the molecular mechanisms of tumour progression [31–35]. In the present study, WGCNA analysis identified a total of 33 lncRNAs in the four stable modules with relevant biological functions and correlation with specific clinical factors of OC. Based on the expression level of these 33 lncRNAs in the TCGA dataset and the clinical prognostic information of the samples, a short-list of 19 lncRNAs were screened using univariate Cox regression analysis. Finally, an optimal prognosis combination containing 5 lncRNAs (GAS5, HCP5, PART1, SNHG11, and SNHG5) was identified using a Cox-PH model. Considering that risk assessment tools can help to detect high-risk populations for a disease [15], the present study establishes a new risk assessment system based on the above prognostic gene signature. The effectiveness of the RS model was tested in both the training set and validation sets, and the results indicated that the risk assessment tool could successfully distinguish a population at high risk of future OC development. This method is simple and inexpensive enough to be used in normal clinical practice and mass screening. Compared with the previous lncRNA signatures and RS models for OC [3, 6, 9, 19], the present prognostic lncRNA combination and RS model may be more reliable because they were screened based on the WGCNA analysis instead of single differential gene analysis.

The five prognostic genes have all been reported to be associated with human cancer, and three (GAS5, HCP5, and SNHG11) have reported associations with OC. GAS5 (growth arrest-specific transcript 5) was originally isolated from NIH 3 T3 cells using subtraction hybridization [36]. The latest studies demonstrated that GAS5 usually functions as a tumour suppressor to control apoptosis of various cancer cells, including breast cancer, prostate cancer, renal cell carcinoma, and ovarian cancer [12, 37–39]. Furthermore, GAS5 acts as tumour suppressor and has been suggested as a potential target for diagnosis and therapy of OC [12]. HCP5 (HLA Class I Histocompatibility Antigen Protein P5) is localised in the major histocompatibility complex (MHC) class I region and has involvement in the development of various tumours including OC [40–43]. SNHG11 (small nucleolar RNA host gene 11) is an obesity-associated lncRNA, and is involved in positive regulation of cell proliferation in OC [44]. PART-1 (prostate androgen regulated transcript 1) is a gene known to be predominantly expressed in the prostate- and androgen-regulated. Its aberrant expression has been associated with poor prognosis of prostate cancer, non-small cell lung cancer, and colorectal cancer, leading to the suggestion of its use as a novel tumour marker [45–47]. SNHG5 (small nucleolar RNA host gene 5) has been strongly implicated in cancer-related processes, such as cell differentiation, cell proliferation, and metastasis [48–50]. The strong evidence of the association of all five lncRNAs with cancer and/or OC supports the conclusion that this study identified potential biomarkers for predicting the prognosis of OC patients, which should also help future research into the pathogenesis of OC.

It has been demonstrated that lncRNAs play important roles in a variety of biological processes by regulating target genes at transcriptional, posttranscriptional and epigenetic levels [51, 52]. Therefore, we investigated the target genes regulated by the 5 prognostic lncRNAs to decipher their potential biological function in the pathogenesis of OC. The result of pathway enrichment analysis showed that the network modules related to the five prognostic genes were significantly associated with cell local adhesion, cancer signalling pathways, JAK-STAT signalling, and endogenous cell receptor interaction. According to functional analyses of lncRNA regulators, it was found that low expression of GAS5 could promote proliferation, metastasis, and infiltration of OC cells, and as a result, was considered to be associated with poor prognosis of OC [11, 12]. Most of the mRNAs in the regulated pathway are associated with the development of OC. For example, up-regulation of RASSF5 expression can inhibit the growth of OC cells [53], over-expression of FZD3 can increase the survival time of OC patients [54], and inhibition of MMP9 gene expression can block metastasis of ovarian cancer cells [55]. Therefore, the five prognostic-related lncRNAs identified in the present study may play roles in the initiation and development of OC by regulating genes involved in cell adhesion and the JAK-STAT signalling pathway.

Although the independent validation performed in this study and the results of previous reports both indicate that the present model should be effective, there are limitations of the present study. Primarily, as this was an extensive bioinformatics study based on previously published data, our results should be further validated using in vitro and in vivo models. However, our results form a strong basis for other researchers to carry out the relevant future research.

## Conclusions

In conclusion, our study constructed a co-expression network and excavated four modules with specific biological functions related to OC. A risk assessment tool for predicting prognosis of OC was further identified and validated based on the expression of 5 prognostic genes. The present risk assessment tool could provide a novel reliable method to identify individuals at high risk of OC, and the 5 prognostic genes could be promising prognostic biomarkers for OC.

## Data Availability

The datasets used and/or analyzed during the current study are available from TCGA database (https://gdc-portal.nci.nih.gov/) and GEO database (http://www.ncbi.nlm.nih.gov/geo/) with assession number of GSE32062 [56] (https://www.ncbi.nlm.nih.gov/geo/query/acc.cgi?acc=GSE32062), GSE17260 [57] (https://www.ncbi.nlm.nih.gov/geo/query/acc.cgi?acc=GSE17260), GSE49997 [58] (https://www.ncbi.nlm.nih.gov/geo/query/acc.cgi?acc=GSE49997), GSE26712 [59, 60] (https://www.ncbi.nlm.nih.gov/geo/query/acc.cgi?acc=GSE26712) and GSE31245 [61] (https://www.ncbi.nlm.nih.gov/geo/query/acc.cgi?acc=GSE31245). Processed data are available from the corresponding author on reasonable request.

## Abbreviations

CESC: Cervical cancer
CVL: Cross-validation likelihood
ES: Enrichment score
GSEA: Gene set enrichment analysis
lncRNAs: Long non-coding RNAs
NES: Normalized enrichment score
OC: Ovarian cancer
RS: Risk score
WGCNA: Weighted gene co-expression network analysis
